# Evaluation of Different Approaches for Pain Management in Postoperative General Surgery Patients: A Comprehensive Review

**DOI:** 10.7759/cureus.48573

**Published:** 2023-11-09

**Authors:** Yashraj Jain, Ranjana Lanjewar, Yashwant Lamture, Dushyant Bawiskar

**Affiliations:** 1 General Surgery, Jawaharlal Nehru Medical College, Datta Meghe Institute of Higher Education and Research, Wardha, IND; 2 Sports Medicine, Abhinav Bindra Sports Medicine and Research Institute, Bhubaneswar, IND

**Keywords:** recovery, analgesia, surgery, non-opioids, opioids, nsaid

## Abstract

The treatment of postoperative pain is a crucial component of postoperative patient care. To reduce discomfort, encourage early mobilisation, reduce complications, and improve overall recovery, effective pain management is crucial. To maximise pain relief and reduce the need for opioids and their accompanying adverse effects, a multimodal analgesic strategy, combining several drugs and procedures, is frequently used. Opioids continue to be a crucial part of postoperative pain treatment for moderate to severe pain; however, due to potential side effects and dependency concerns, their usage should be carefully controlled. Non-opioid analgesics, such as non-steroidal anti-inflammatory medications (NSAIDs) and paracetamol, are widely used as a component of a multimodal regimen and play an important role in pain management. Postoperative pain management ultimately aims to deliver efficient analgesia, enhance patient comfort, and foster a speedy recovery while minimising the dangers and adverse effects connected with pain medicines. To create a thorough and unique pain management strategy for each patient, close coordination between the surgical team, anesthesiologists, and pain management specialists is essential.

## Introduction and background

A broad variety of illnesses, traumas, and disorders can be surgically treated as part of the medical specialty known as general surgery. General surgeons are skilled medical professionals with the expertise and abilities to operate on the abdomen, breasts, skin, digestive tract, endocrine system, and other regions of the body. They frequently act as the main surgeons in charge of diagnosing, monitoring, and treating surgical disorders. Both elective and urgent operations fall under the general surgery umbrella. To treat non-life-threatening ailments such as hernias, gallbladder illnesses, appendicitis, and different benign tumors, elective procedures are scheduled in advance. On the other side, urgent procedures are done to cure life-threatening disorders such as trauma, perforations, bleeding, and infections. The whole surgical care process, including preoperative diagnosis and evaluation, surgical intervention, postoperative care, and follow-up, is handled by general surgeons. To offer thorough patient care, they collaborate closely with other medical professionals such as anaesthesiologists, surgical assistants, nurses, and experts in relevant disciplines [1-4].

With improvements in surgical methods, technology, and less invasive techniques, the field of general surgery is constantly changing. General surgeons can perform open surgery 'the conventional way' or use minimally invasive techniques that need fewer incisions and specialized equipment, such as laparoscopy or robotic surgery. Patients frequently experience shorter hospital stays, less postoperative discomfort, and speedier recovery periods because of these less invasive treatments. General surgeons offer crucial non-operative care in addition to their surgical expertise, such as the management of acute and chronic illnesses, wound care, and counselling patients on lifestyle changes and preventative measures. They collaborate with experts in disciplines including cancer, gastroenterology, vascular surgery, and more to coordinate multidisciplinary treatment for patients. The field of general surgery covers a wide variety of surgical techniques and is varied and dynamic.

General surgeons are prepared to manage a range of surgical situations, from simple emergencies to complicated cases. Through continual research and innovation, they are dedicated to providing high-quality surgical treatment, enhancing patient outcomes, and advancing surgical methods and expertise [5-8].

Surgery can result in varied degrees of tissue stress and acute pain, which can negatively impact a patient's comfort, well-being, and healing. To reduce suffering, encourage early mobilisation, ease rehabilitation, and avoid problems linked to insufficient pain control, effective pain management is crucial. Postoperative pain treatment aims to minimise negative effects and promote quick recovery while providing the best possible pain relief. It incorporates a multimodal strategy that integrates several methods and interventions catered to the requirements of the specific patient. A thorough pain management strategy should take into account the patient's overall health, the surgery, the anticipated length of pain (in chronic cases which is approximately two to three months), and the potential dangers and advantages of various therapies.

The use of a multimodal analgesic strategy is one of the cornerstones of postoperative pain treatment. In order to address pain pathways at various levels, this strategy entails administering a number of analgesic drugs with varied modes of action. It is possible to provide greater pain relief with lower dosages of each medicine by synergizing pharmaceuticals from other groups, such as opioids, non-steroidal anti-inflammatory drugs (NSAIDs), paracetamol, and local anaesthetics. This lowers the risk of adverse effects. Adverse postoperative pain is frequently treated with opioids like morphine. They exert analgesia by acting on the central nervous system, but they also have negative side effects including drowsiness, respiratory depression, constipation, and the possibility of addiction [9]. Therefore, opioid-sparing procedures are frequently used to give focused pain relief while lowering the need for systemic opioids. These approaches include the use of regional anaesthesia techniques, such as epidural or peripheral nerve blocks. Multimodal analgesia relies heavily on non-opioid analgesics such NSAIDs and paracetamol. Prostaglandins, which are inflammatory mediators involved in pain signalling, are produced by prostaglandin inhibition, which is how NSAIDs work to reduce inflammation [10-13]. Acetaminophen commonly known as paracetamol affects how the central nervous system perceives pain. Both kinds of drugs have analgesic effects and can be administered to individuals with mild to moderate pain either alone or in combination with opioids.

Enhanced recovery after surgery (ERAS) protocols, which aim to improve perioperative care and speed up recovery, have received more attention in recent years. A crucial part of ERAS protocols is pain management, and techniques such as preoperative education, proactive analgesia, and early oral medicine intake are used to enhance pain management and patient outcomes [14]. Healthcare professionals may successfully control pain while minimising side effects and encouraging early recovery by combining several analgesic approaches, including opioids, non-opioids, and regional anaesthesia. The implementation of improved recovery regimens improves patient outcomes and pain management even more. To offer the best pain relief and enhance the surgical experience overall, customised pain management regimens based on patient traits and surgical techniques are required [15-18].

## Review

Methodology

A comprehensive and detailed strategy was followed while researching “Postoperative pain management”. The results were chosen from research databases all over the world. Research databases like PubMed, Google Scholar, MEDLINE, Embase etc. were researched thoroughly with keywords like “NSAID”, “Opioids”, and “Analgesia”. Articles in languages other than English were filtered out and not considered. Editor’s note was also filtered out. The articles chosen were published from the year 2000 till 30-10-2023.

Discussion

General surgery covers a vast spectrum of operations in medical anomalies and illnesses. All these surgeries require a comprehensive pain management strategy which involves several aspects to ease the suffering and increase the quality of living. For example in abdominal surgery, postoperative pain control is essential for enhancing patient comfort, maximizing healing, and avoiding complications. Appendectomy, cholecystectomy, hernia repair, and bowel resection are just a few of the numerous treatments that fall under the umbrella of abdominal surgery. Depending on the surgery, the patient, and the surgeon's preferences, the method of pain management may change. Commonly used pain management techniques include opioid-based analgesia, multimodal analgesia, local anaesthetic techniques, epidural analgesia, regional nerve blocks, Enhanced Recovery After Surgery (ERAS) protocols, patient-controlled analgesia, etc.

Nowak et al. concluded in a study, which was focused on the usage of general anaesthesia in post-operative pain management, that a safe, practical, affordable, non-drug method to lessen surgical pain and opioid consumption may be provided by therapeutic recommendations broadcast through earbuds under general anaesthesia, with the potential for more widespread application [12].

In analgesia based on opioids in the immediate postoperative period, opioids, such as morphine or oxycodone, are frequently used for initial pain relief. Patients can self-provide modest amounts of opioids as needed when using patient-controlled analgesia (PCA) pumps to administer intravenous (IV) opioids. It's crucial to keep an eye out for opioid-related side effects including respiratory depression and drowsiness. To lessen the need for opioids and lessen adverse effects, multimodal analgesia, which mixes various analgesic classes, is promoted. Acetaminophen and non-steroidal anti-inflammatory medications (NSAIDs) are two non-opioid analgesics that are frequently used in conjunction with opioids. Acetaminophen operates on the central nervous system (CNS) to reduce pain perception, whereas NSAIDs reduce inflammation and block pain signalling pathways. Targeted pain management can be achieved and the requirement for systemic opioids reduced by local anaesthetic infusion at the surgical site. Techniques like field blocks with long-acting local anaesthetics or local wound infiltration may be used [19-22].

Basat et al. mentioned in a study in which results showed that blocking two nerves via an arthroscopy technique was an effective way to control pain during the healing process. As a result, patients' comfort may increase and they could recover quickly [23].

The administration of local anaesthetics can be made more precise and effective by using ultrasound-guided procedures. By inserting a catheter into the epidural area, local anaesthetics and/or opioids are continuously infused into the patient during epidural analgesia. Excellent pain management is provided via epidural analgesia, which is particularly useful for big abdominal procedures. It necessitates close observation for any potential side effects, including hypotension, urine incontinence, and respiratory depression. ERAS procedures prioritise perioperative care optimisation to speed up recovery. The foundation of ERAS for abdominal surgery is preoperative education, standardised analgesic regimes, and early mobilisation.

When necessary, minimally invasive procedures can lessen postoperative discomfort and hasten recovery. Patients who use PCA pumps can self-administer modest intravenous dosages of opioids for on-demand pain management. Patients can individualise their analgesic needs and actively engage in pain treatment thanks to PCA. It is essential to closely monitor usage guidelines and any negative effects [24-27].

Enhanced recovery after surgery (ERAS)

The goal of Enhanced Recovery After Surgery (ERAS), commonly referred to as "fast-track" or "accelerated recovery" procedures, is to improve patient outcomes and hasten recovery after surgery. It is a multimodal, evidence-based approach to perioperative treatment. In order to improve patient recovery, lower complications, and shorten hospital stays, ERAS programmes use a complete range of perioperative treatments, including preoperative, intraoperative, and postoperative methods. Abdominal, orthopaedic, colorectal, and gynaecological operations are just a few of the surgical specialities that have adopted the concept and are in wide use of ERAS concepts. Ileus is a brief absence of the intestines' regular muscular contractions. Common reasons include drugs that affect the intestinal motility and abdominal surgery. Ileus causes bloating, vomiting, constipation, cramping, and appetite loss. X-rays are used to make the diagnosis. Patient education and preoperative optimisation of the process is very important. In order to improve surgical preparedness, the patient's health status should be evaluated and optimised before surgery. Patients should be made aware regarding outcomes of the therapeutics, which can aid the healing process. This will make sure the patient has no unreasonable expectation. To reduce surgical risks, one should stop smoking, maintain a healthy weight, and, if necessary, regulate their blood sugar [28-31].

Optimal postoperative care aims for minimal complications, shortened hospital stays, and effective pain management. This includes a mix of pain relievers like opioids, non-opioids, and regional anaesthesia to minimize opioid-related side effects. Initiating pain management before surgery helps prevent central sensitization. Encouraging early resumption of oral intake, including clear fluids and solid food, promotes faster recovery, reduces the risk of postoperative ileus, and supports gastrointestinal health [23,32,33].

Opioids in post-operative pain management

Opioids are frequently used to produce analgesia and alleviate moderate to severe pain after surgical operations in postoperative pain management. Although they have possible hazards and adverse effects, they are useful in treating acute pain. Opioids affect how pain is perceived by attaching to opioid receptors in the central nervous system, which slows down the transmission of pain signals. However, they can also interact with kappa and delta receptors, resulting in analgesia and other effects. They predominantly operate on mu-opioid receptors. Opioids provide potent pain relief and are effective in controlling acute pain after surgery. They can improve patient comfort, promote resting, and aid in the facilitation of early mobilization and rehabilitation. Adequate pain control with opioids can reduce the physiological stress response, improve respiratory function, and enhance patient recovery and outcome.

Sedation, respiratory depression, nausea, vomiting, constipation, urine retention, itching, and cognitive impairment are just a few of the negative effects that opioids can have. It is important to regularly monitor individuals using opioids, especially if they have respiratory depression, especially in the days right after surgery. Long-term or high-dose opioid usage may increase one's chance of developing dependence, addiction, and an opioid use disorder. However, when opioids are properly given for acute pain treatment following surgery, the risk is comparatively modest. Patients' sensitivity to and responses to opioids might vary, making individualised treatment and constant observation necessary. Elderly and opioid-related side effects and consequences may be more likely to affect older persons and people with comorbid conditions [34-36]. There should be vigilance and dosage changes.

Opioid-sparing tactics are frequently used to cut down on usage and minimise adverse effects due to the possible hazards linked with opioids. NSAIDs, paracetamol, and adjuvant medicines can be used in conjunction with opioids to improve pain management while lowering opioid dosages. Regional anaesthesia, methods like peripheral nerve blocks and epidural analgesia can give localised pain relief while lowering the need for systemic opioids. Patient-controlled analgesia (PCA) pumps enable patients to regulate their own pain while consuming less opioids overall by allowing them to self-administer modest dosages of the drugs within the specified ranges. Based on the patient's particular features, the surgical process, and the level of discomfort, pain treatment should be tailored. To calculate the proper opioid dosage, take other analgesics into consideration, and keep an eye out for negative effects, healthcare practitioners evaluate patients' pain levels, medical histories, and risk factors.

Non-opioids in post-operative pain management

Non-opioid analgesics are essential for managing postoperative pain, both on their own and in combination with other analgesics. These drugs can effectively treat pain while reducing the dangers and negative effects related to opioids. Because of their potent analgesic and anti-inflammatory effects, NSAIDs including ibuprofen, diclofenac, and ketorolac are often utilised. They prevent the synthesis of prostaglandins, which are responsible for inflammation and discomfort. NSAIDs are especially helpful for pain brought on by inflammation, such as that experienced following orthopaedic or abdominal surgery. When administering NSAIDs to individuals who have a history of gastrointestinal bleeding, renal insufficiency, or cardiovascular illness, caution is suggested.

A popular and regularly used non-opioid analgesic is paracetamol. Although it has only weak anti-inflammatory actions, it centrally regulates pain perception and temperature. Acetaminophen is frequently used in conjunction with other analgesics and is helpful for treating mild to severe postoperative pain. When taken in accordance with the authorised dosages, it has a favourable safety profile, but excessive or extended usage might result in liver damage. The term "synergism" describes the improved analgesic or pain-relieving effect that results from the combination of paracetamol (acetaminophen) and nonsteroidal anti-inflammatory medicines (NSAIDs), as opposed to their individual usage. Their various modes of action combine to provide a synergy that may help with pain control.

A subclass of NSAIDs known as COX-2 inhibitors, including celecoxib, preferentially block the enzyme cyclooxygenase-2, which is implicated in pain and inflammation [37-40]. A family of drugs known as cyclooxygenase-2 (COX-2) inhibitors specifically targets and inhibits the cyclooxygenase-2 enzyme. These medications are intended to lessen inflammation and discomfort. Prostaglandins (PG) are lipid molecules that are important for many physiological processes, including inflammation, pain, and blood flow control. One of the enzymes involved in PG production is COX-2. Prostaglandins (PG) are a class of lipid molecules with a variety of physiological functions. Pro-inflammatory prostaglandins are generated by COX-2, whereas prostaglandins produced by COX-1, in particular, protect the stomach lining and support a number of physiological functions. Compared to non-selective NSAIDs, these drugs offer analgesia with maybe less gastrointestinal side effects. Patients who are more susceptible to gastrointestinal issues or who cannot take conventional NSAIDs may benefit most from COX-2 inhibitors. Tramadol is a special drug that works through both opioid and non-opioid processes. It decreases the reuptake of norepinephrine and serotonin and functions as a mild mu-opioid receptor agonist. In some circumstances, tramadol could be used instead of heavier opioids to treat moderate pain. Due to the possibility of adverse effects, such as drowsiness, nausea, and the danger of serotonin syndrome, caution is suggested when using tramadol [41-44]. The improved analgesic or pain-relieving effect that results from taking tramadol and paracetamol (acetaminophen) combined over their separate uses is referred to as their synergy. This combination can offer more effective pain relief and is frequently given for moderate to severe pain.

For postoperative pain management, local anaesthetics like lidocaine or bupivacaine can be utilised in a variety of ways. Targeted pain management can be achieved by injecting local anaesthetics into the surgical site or using catheters in the wound. Local anaesthetics are used to block pain signals coming from certain regions when using regional anaesthesia procedures like peripheral nerve blocks or epidural analgesia. These methods can effectively manage pain and perhaps lessen the requirement for systemic analgesics like opioids.

Pregabalin and gabapentin are anticonvulsants that also have analgesic effects. They may be helpful in surgical procedures linked to nerve damage or neuropathic pain syndromes and can help alleviate neuropathic pain. Gabapentinoids are frequently used as supplements to other analgesics and can lessen the need for opioids.

Pregabalin and gabapentin are two gabapentinoids that have demonstrated effectiveness in the treatment of postoperative pain, especially in situations of neuropathic pain or operations requiring nerve damage. The release of excitatory neurotransmitters is decreased by gabapentinoids because they bind to the alpha-2-delta subunit of voltage-gated calcium channels in the central nervous system. They can have analgesic effects by regulating the transmission of pain signals, notably for neuropathic pain.

Relief from Neuropathic Pain

Gabapentinoids have been useful in reducing neuropathic pain, which can develop after a surgically caused nerve lesion. Gabapentinoids may lessen the requirement for greater opioid dosages by enhancing the analgesic effects of opioids or other non-opioid analgesics, possibly minimising opioid-related adverse effects and problems. Gabapentinoids can be used in conjunction with other analgesics to increase overall pain management and raise patient comfort.

Preoperative Dosing

Some studies have found that beginning gabapentinoids before surgery (preemptive analgesia) is more effective than starting them after surgery.

Dosage

Depending on the patient and the surgery, different gabapentinoids have different optimum doses. A loading dosage is often followed by maintenance doses for a certain amount of time.

Individualised Approach

The gabapentinoid treatment dose and duration should be adapted to the individual patient's requirements, taking into account elements including renal function, co-occurring conditions, and probable adverse effects. Sedation and dizziness are frequent adverse effects of gabapentinoids, especially when used in larger dosages. These side effects should be mentioned to patients, especially while operating machinery or operating a vehicle. Some people who use gabapentinoids may develop disorientation or a decline in cognitive function.

Renal Function

Since gabapentinoids are mostly eliminated through the kidneys, individuals with compromised renal function may require dose changes. The risk of sedation and respiratory depression can be raised when gabapentinoids are used with other drugs such as opioids and CNS depressants. Different people may react differently to gabapentinoids. While some people may only have a modest reaction, others may receive tremendous pain alleviation. Monitoring is necessary to inform therapy modifications and guarantee effective pain management. This includes regular evaluation of pain intensity, side effects, and overall response to gabapentinoids [45-48].

Analgesia and its effectiveness

Analgesia is the term used to describe the use of different drugs and treatment methods to reduce pain and offer respite to patients following surgery. For the comfort of the patient, quick recovery, fewer problems, and effective postoperative analgesia, opioids such as morphine, oxycodone, or fentanyl are strong analgesics frequently used for moderate to severe postoperative pain. They can be given via mouth, intravenous, intramuscular, or patient-controlled analgesia (PCA) pumps, among other delivery methods. Opioids offer efficient pain relief but can also cause drowsiness, respiratory depression, constipation, and nausea, among other undesirable side effects. Ibuprofen, diclofenac, and ketorolac are examples of NSAIDs, which are analgesics with anti-inflammatory characteristics. They do this by preventing the synthesis of prostaglandins, which are responsible for both pain and inflammation. NSAIDs are especially useful for pain brought on by inflammation, such as that experienced following orthopaedic or abdominal procedures. When administering NSAIDs to individuals with a history of gastrointestinal bleeding, renal insufficiency, or cardiovascular illness, caution should be used. Table 1 shows a brief conclusion from the selected review articles.

**Table 1:** List of studies included in the review

Author	Year	Type	Conclusion
Chowdhury et al. [49]	2019	Randomized control trial	Injectable dosage of Bupivacaine was not completely effective in post-operative pain management.
Kinoshita et al. [50]	2019	Randomized control trial	Thoracic epidural analgesia along with acetaminophen works better than thoracic epidural analgesia (TEA) alone in post-operative pain management.
Nowak et al. [12]	2020	Multicentre randomized control trial	Non-drug interventions have shown remarkable progress in post-operative pain management.
Khan et al. [51]	2023	Randomized control trial	Dexamethasone has proven to be effective in post-operative pain management among patients after total knee arthroplasty.
Boules et al. [13]	2016	Review article	Flexible endoscopy has developed into a crucial technique for treating patients undergoing bariatric surgery. The advantage of endoscopy is that it may be used for both therapeutic and diagnostic purposes.
Sierzantowicz et al. [15]	2020	Original article	The way a patient was admitted for surgery had a big impact on how much pain they felt.
Hubner et al. [24]	2020	Article	The majority of the data in this area of surgery is indirect and poor. It is wise to apply these suggestions with caution while prospectively evaluating their viability and outcomes in standard clinical practice.

## Conclusions

Effective postoperative pain management, crucial for patient comfort and recovery, involves a multimodal approach. This includes combining various analgesics to minimize risks and side effects. Opioids are effective but should be used cautiously due to potential adverse effects. Non-opioid options like NSAIDs and paracetamol can be used alone or with opioids. Regional anesthesia techniques, like nerve blocks, can reduce the need for systemic opioids. Adjuvant medications may be considered for neuropathic pain or when opioids alone are insufficient. Tailoring treatment to individual factors, including age, comorbidities, and the surgical procedure, is essential. Continuous monitoring is required to adjust treatment regimens and ensure effective pain management.

## References

[1] Looi YC, Audisio RA (2007). A review of the literature on post-operative pain in older cancer patients. Eur J Cancer.

[2] Behrns KE, Wexner SD (2021). Artificial intelligence: not an oxymoron in surgery. Surgery.

[3] Mwachiro M, Mwachiro E, Wachu M, Koske W, Thure L, Parker RK, White RE (2020). Assessing post-operative pain with self-reports via the Jerrycan pain scale in rural Kenya. World J Surg.

[4] Taylor I (1998). Calmanization of general surgery. J R Soc Med.

[5] Orhurhu V, Orman S, Peck J (2020). Carpal tunnel release surgery- A systematic review of open and endoscopic approaches. Anesth Pain Med.

[6] Marcadis AR, Spencer T, Sleeman D, Velazquez OC, Lew JI (2020). Case distributions in general surgery residency: subspecialization occurs before fellowship. Surgery.

[7] Wang ZQ, Zhan SY, Fransen M, Lin JH (2012). Clinical attitudes towards pain treatment post-orthopedic surgery: a multicenter study in Beijing. Chin Med J (Engl).

[8] Manchikanti L, Singh V, Pampati V, Boswell MV, Benyamin RM, Hirsch JA (2009). Description of documentation in the management of chronic spinal pain. Pain Physician.

[9] Schwenk ES, Mariano ER (2018). Designing the ideal perioperative pain management plan starts with multimodal analgesia. Korean J Anesthesiol.

[10] Williams MD, Grunvald MW, Skertich NJ, Hayden DM, O'Donoghue C, Torquati A, Becerra AZ (2021). Disruption in general surgery: randomized controlled trials and changing paradigms. Surgery.

[11] Kumar TS, Muthuraman M, Krishnakumar R (2014). Effect of the raga ananda bhairavi in post operative pain relief management. Indian J Surg.

[12] Nowak H, Zech N, Asmussen S (2020). Effect of therapeutic suggestions during general anaesthesia on postoperative pain and opioid use: multicentre randomised controlled trial. BMJ.

[13] Boules M, Chang J, Haskins IN (2016). Endoscopic management of post-bariatric surgery complications. World J Gastrointest Endosc.

[14] Melnyk M, Casey RG, Black P, Koupparis AJ (2011). Enhanced recovery after surgery (ERAS) protocols: time to change practice?. Can Urol Assoc J.

[15] Sierżantowicz R, Lewko J, Bitiucka D, Lewko K, Misiak B, Ładny JR (2020). Evaluation of pain management after surgery: an observational study. Medicina (Kaunas).

[16] Beverly A, Kaye AD, Ljungqvist O, Urman RD (2017). Essential elements of multimodal analgesia in enhanced recovery after surgery (ERAS) guidelines. Anesthesiol Clin.

[17] Al Samaraee A, Rhind G, Saleh U, Bhattacharya V (2010). Factors contributing to poor post-operative abdominal pain management in adult patients: a review. Surgeon.

[18] Bass BL (2007). Fundamental changes in general surgery residency training. Am Surg.

[19] Fernández-Cruz L (2004). General surgery as education, not specialization. Ann Surg.

[20] Downs AR (1982). General surgery. Arch Surg.

[21] Potts JR lll (2019). General surgery residency: past, present, and future. Curr Probl Surg.

[22] Leppäniemi A (2011). General surgery--a vision for the future. Scand J Surg.

[23] Basat HÇ, Uçar DH, Armangil M, Güçlü B, Demirtaş M (2016). Post operative pain management in shoulder surgery: suprascapular and axillary nerve block by arthroscope assisted catheter placement. Indian J Orthop.

[24] Hübner M, Kusamura S, Villeneuve L (2020). Guidelines for Perioperative Care in Cytoreductive Surgery (CRS) with or without hyperthermic IntraPEritoneal chemotherapy (HIPEC): Enhanced Recovery After Surgery (ERAS®) Society Recommendations - Part II: postoperative management and special considerations. Eur J Surg Oncol.

[25] Griffen WO Jr (1991). General surgery, a true specialty. Surgery.

[26] Maruthappu M, Sharma A, Shalhoub J, Davies A (2011). General surgery: allow its extinction or begin its revival?. Br J Hosp Med (Lond).

[27] Mateo Vallejo F (2012). General surgery: present and future. Int J Surg.

[28] Chou R, Gordon DB, de Leon-Casasola OA (2016). Management of postoperative pain: a clinical practice guideline from the American Pain Society, the American Society of Regional Anesthesia and Pain Medicine, and the American Society of Anesthesiologists' Committee on Regional Anesthesia, Executive Committee, and Administrative Council. J Pain.

[29] Vitale SG, Alonso Pacheco L, Haimovich S (2021). Pain management for in-office hysteroscopy. A practical decalogue for the operator. J Gynecol Obstet Hum Reprod.

[30] Gacio MF, Lousame AM, Pereira S, Castro C, Santos J (2016). Paravertebral block for management of acute postoperative pain and intercostobrachial neuralgia in major breast surgery. Braz J Anesthesiol.

[31] Coleman AC (2017). Perioperative pain management for upper extremity surgery. Orthop Clin North Am.

[32] Chierici A, Frontali A (2021). Post-hemorrhoidectomy pain management: the latest news. Rev Recent Clin Trials.

[33] Ocitti EF, Adwok JA (2000). Post-operative management of pain following major abdominal and thoracic operations. East Afr Med J.

[34] Jensen B (2012). Post-operative pain and pain management in children after dental extractions under general anaesthesia. Eur Arch Paediatr Dent.

[35] Sama HD, Bang'na Maman AF, Djibril M, Assenouwe M, Belo M, Tomta K, Chobli M (2014). Post-operative pain management in paediatric surgery at Sylvanus Olympio University Teaching Hospital, Togo. Afr J Paediatr Surg.

[36] Butler C, Mmonu N, Cohen AJ, Rios N, Huang CY, Breyer BN (2022). Pre-operative assessment tool to predict post-operative pain and opioid use in outpatient urologic surgery. Urology.

[37] Behman R, Cleary S, McHardy P (2019). Predictors of post-operative pain and opioid consumption in patients undergoing liver surgery. World J Surg.

[38] Yunus AA, Nwasor EO, Idris ME, Ejagwulu FS (2012). Regional analgesia for post-operative pain management--initial experience in a low resource setting. East Afr Med J.

[39] Mukerjee S, Gupta T (1997). Surgery in India. Arch Surg.

[40] Hamberger B (1998). Surgery in Sweden. Arch Surg.

[41] (2018). The ethics of surgery. JAMA.

[42] Wilkinson N, Zuckerman R (2020). Surgical oncology for the general surgeon. Surg Clin North Am.

[43] Douleh DG, Chambers L, Parry JA (2022). The effect of regional anesthesia blocks on post-operative pain after ambulatory orthopedic trauma surgery. Eur J Orthop Surg Traumatol.

[44] Jordan GL Jr (1991). The future of general surgery. Am J Surg.

[45] Wickham JE (1987). The new surgery. Br Med J (Clin Res Ed).

[46] Grabenwöger M (2016). The power of surgery. Eur J Cardiothorac Surg.

[47] Devon K (2015). The practice of surgery. Narrat Inq Bioeth.

[48] Jones AT, Barry CL, Ibáñez B, Buyske J (2021). Using multiple modes of assessment in general surgery for board certification. Am J Surg.

[49] Chowdhury TS, Mustary M, Chowdhury TA (2023). A randomized controlled trial depicting postoperative pain score following port-site infiltration during laparoscopy. J South Asian Fed Obstet Gynecol.

[50] Kinoshita J, Fushida S, Kaji M (2019). A randomized controlled trial of postoperative intravenous acetaminophen plus thoracic epidural analgesia vs. thoracic epidural analgesia alone after gastrectomy for gastric cancer. Gastric Cancer.

[51] Khan J, Ashraf RA, Bilal Shabbir HM (2023). The effect of dexamethasone on postoperative pain management in patients undergoing total knee arthroplasty: a randomized controlled trial. Cureus.

